# Whole exome sequencing uncovered highly penetrant recessive mutations for a spectrum of rare genetic pediatric diseases in Bangladesh

**DOI:** 10.1038/s41525-021-00173-0

**Published:** 2021-02-16

**Authors:** Hosneara Akter, Mohammad Shahnoor Hossain, Nushrat Jahan Dity, Md. Atikur Rahaman, K. M. Furkan Uddin, Nasna Nassir, Ghausia Begum, Reem Abdel Hameid, Muhammad Sougatul Islam, Tahrima Arman Tusty, Mohammad Basiruzzaman, Shaoli Sarkar, Mazharul Islam, Sharmin Jahan, Elaine T. Lim, Marc Woodbury-Smith, Dimitri James Stavropoulos, Darren D. O’Rielly, Bakhrom K. Berdeiv, A. H. M. Nurun Nabi, Mohammed Nazmul Ahsan, Stephen W. Scherer, Mohammed Uddin

**Affiliations:** 1 Genetics and Genomic Medicine Centre, NeuroGen Children’s Healthcare, Dhaka, Bangladesh; 2 Department of Biochemistry and Molecular Biology, University of Dhaka, Dhaka, Bangladesh; 3 Department of Genetic Engineering & Biotechnology, University of Dhaka, Dhaka, Bangladesh; 4 College of Medicine, Mohammed Bin Rashid University of Medicine and Health Science, Dubai, UAE; 5 BioTED, Dhaka, Bangladesh; 6 Department of Child Neurology, NeuroGen Children’s Healthcare, Dhaka, Bangladesh; 7 Department of Endocrinology & Metabolism, Bangabandhu Sheikh Mujib Medical University, Dhaka, Bangladesh; 8 Department of Genetics, Harvard Medical School, Boston, USA; 9 The Centre for Applied Genomics, The Hospital for Sick Children, Toronto, Canada; 10 Translational and Clinical Research Institute, Newcastle University, Newcastle upon Tyne, UK; 11 Genome Diagnostics, Department of Pediatric Laboratory Medicine, The Hospital for Sick Children, Toronto, Canada; 12 Faculty of Medicine, Memorial University, St. John’s, NL Canada; 13 McLaughlin Centre and Department of Molecular Genetics, University of Toronto, Toronto, Ontario Canada

**Keywords:** Medical genomics, Disease genetics

## Abstract

Collectively, rare genetic diseases affect a significant number of individuals worldwide. In this study, we have conducted whole-exome sequencing (WES) and identified underlying pathogenic or likely pathogenic variants in five children with rare genetic diseases. We present evidence for disease-causing autosomal recessive variants in a range of disease-associated genes such as *DHH*-associated 46,XY gonadal dysgenesis (GD) or 46,XY sex reversal 7, *GNPTAB*-associated mucolipidosis II alpha/beta (ML II), *BBS1*-associated Bardet–Biedl Syndrome (BBS), *SURF1*-associated Leigh Syndrome (LS) and *AP4B1*-associated spastic paraplegia-47 (SPG47) in unrelated affected members from Bangladesh. Our analysis pipeline detected three homozygous mutations, including a novel c. 863 G > C (p.Pro288Arg) variant in *DHH*, and two compound heterozygous variants, including two novel variants: c.2972dupT (p.Met991Ilefs*) in *GNPTAB* and c.229 G > C (p.Gly77Arg) in *SURF1*. All mutations were validated by Sanger sequencing. Collectively, this study adds to the genetic heterogeneity of rare genetic diseases and is the first report elucidating the genetic profile of (consanguineous and nonconsanguineous) rare genetic diseases in the Bangladesh population.

## Introduction

Rare genetic disorders, while individually uncommon, collectively impact significant numbers worldwide^1^, with approximately 7,000 rare diseases estimated to affect an estimated 350 million people (European Organization for Rare Diseases; EURORDIS). These disorders encompass a spectrum of rare complex clinical manifestations^2,3^ that can be difficult to diagnose and pinpoint causation. Clinics in developing countries are not equipped with technologically advanced healthcare facilities for diagnosis and treatment. In Bangladesh, genetic tests are not widely used in a clinical setting and mostly applied for medical research purposes^4,5^. The current standard of care does not include first-tier genetic testing in most developing nations. Limited use of targeted (single-gene) sequencing, low-resolution microarray, and karyotype technologies can identify a small fraction of the total population of patients with rare genetic disorders.

Whole exome sequencing (WES) is both an effective and efficient method to detect genetic defects underlying hereditary diseases^6–9^. Diagnoses of rare genetic diseases are essential for improving the health care system of any developing country. Early disease diagnosis can lead to early and targeted therapeutic intervention and can avoid other unnecessary diagnostic investigations. In this study, we performed WES to investigate a series of patients from Bangladesh with rare genetic diseases referred for clinical assessment. Currently, there is no standard of care for rare disorders in Bangladesh. Clinical presentation in these patients was complex^10^, prompting referral for genetic diagnosis. We report seven (7) extremely rare recessive variants that are potentially causal in a range of rare genetic diseases such as, *DHH*-associated 46,XY gonadal dysgenesis (GD) or 46,XY sex reversal 7, *GNPTAB*-associated mucolipidosis II alpha/beta ((ML II), *BBS1*-associated Bardet–Biedl Syndrome (BBS), *SURF1*-associated Leigh Syndrome (LS) and *AP4B1*-associated spastic paraplegia-47 (SPG47)from a small cohort of five unrelated Bangladeshi patients.

## Results

**Fig. 1:**
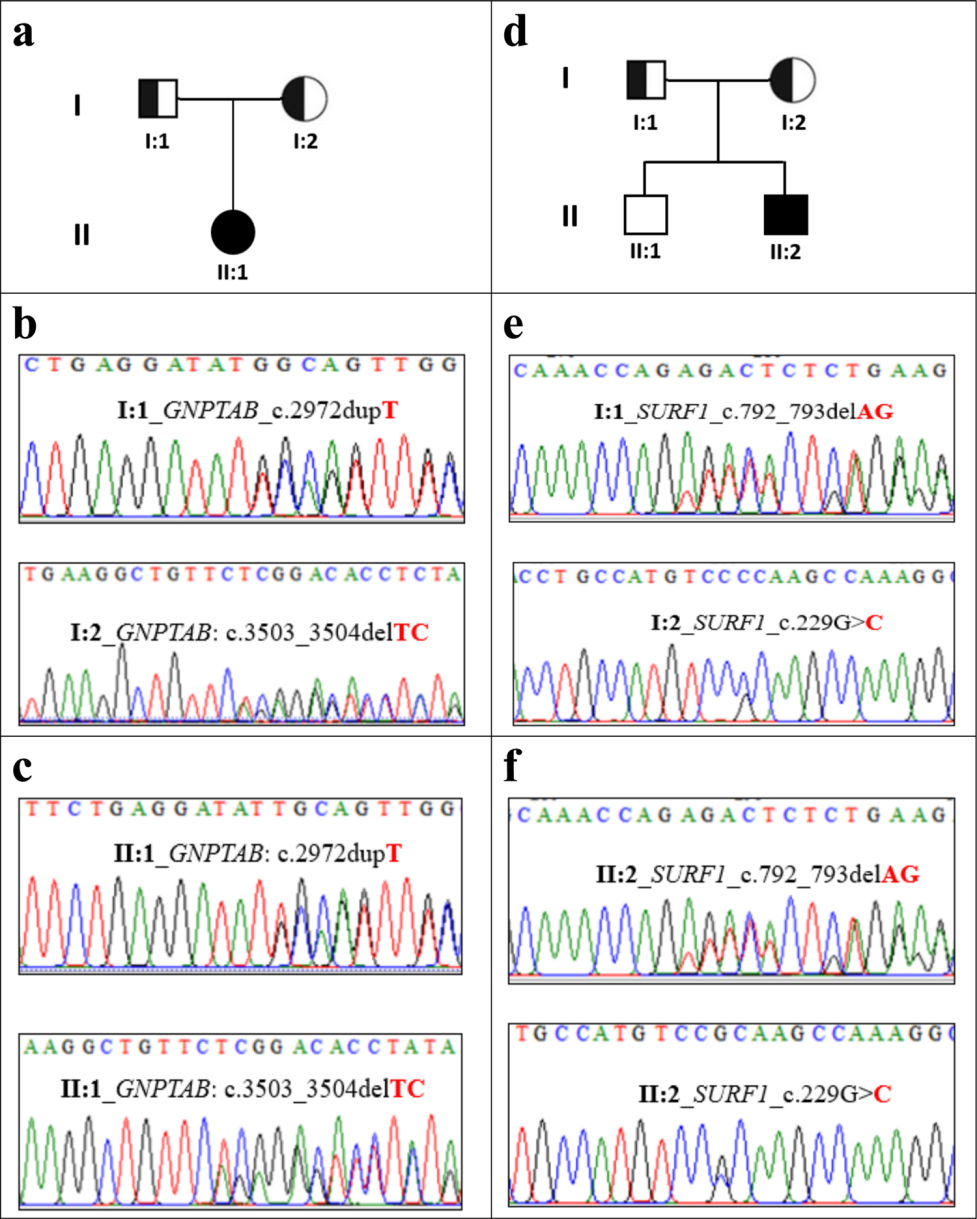
Pedigree and Sanger validation of all compound heterozygous mutations in two unrelated non-consanguineous families (Patient 2 and 4, respectively). **a**, **d** Pedigree of two non-consanguineous families. Square indicates male and circle indicates female. The filled symbols represent affected individuals and partially filled symbols indicate carrier parents. Open symbols indicate healthy individual. **b** Chromatograms from Sanger sequencing of heterozygous *GNPTAB* mutations c.2972dupTand c.3503_3504delTCin father and mother, respectively. **c** Chromatograms from Sanger sequencing of compound heterozygous *GNPTAB* mutations c.2972dupT and c.3503_3504delTCin Patient-2. **e** Chromatograms from Sanger sequencing of heterozygous *SURF1* mutations c.792_793delAGand c.229 G > Cin father and mother, respectively. **f** Chromatograms from Sanger sequencing of compound heterozygous *SURF1* mutations c.792_793delAGand c.229 G > Cin patient-4. His elder brother (II:1) was apparently healthy and was not included in this study.

**Fig. 2:**
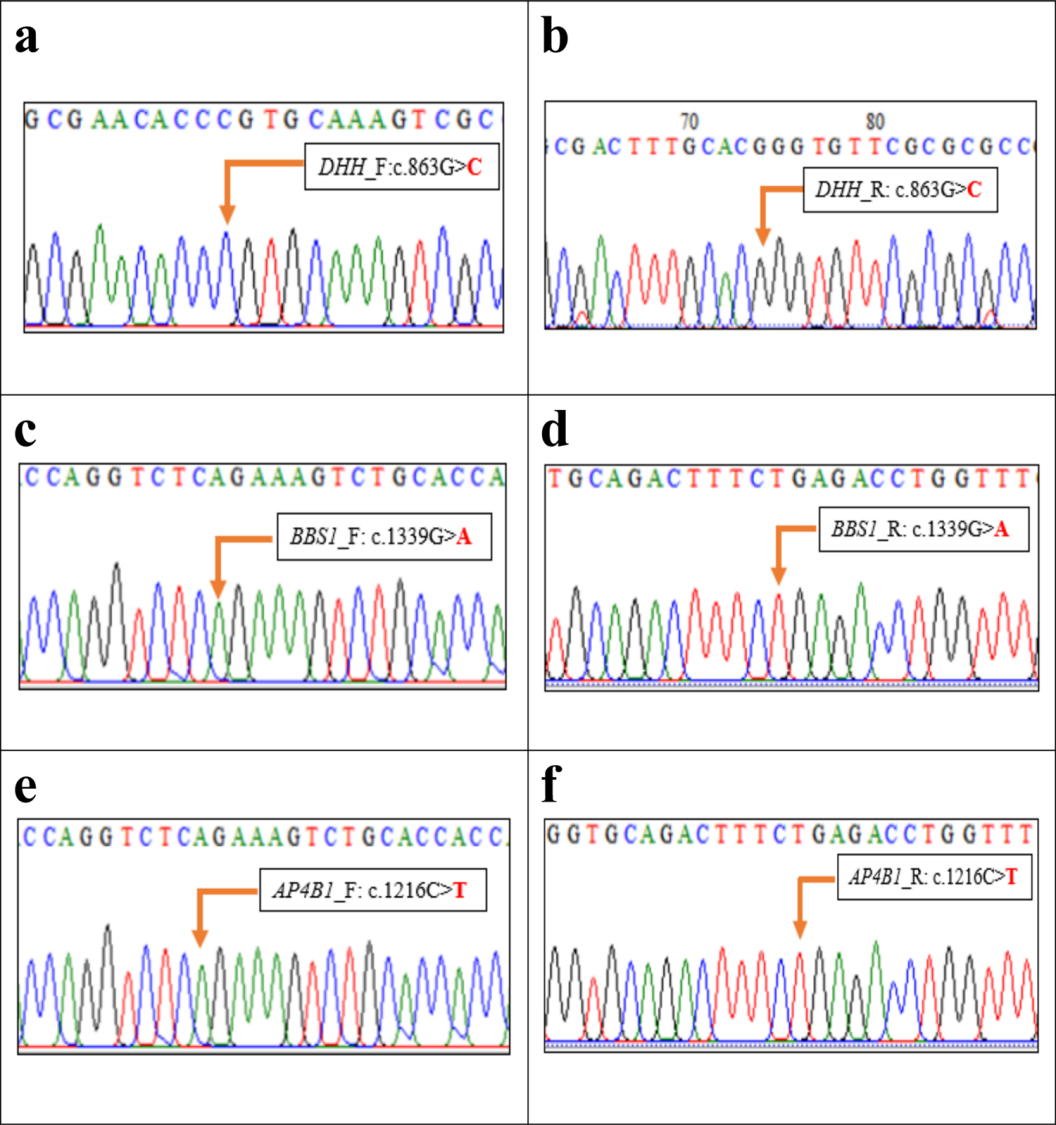
Sanger validation of all homozygous mutations in *DHH*, *BBS1* and *AP4B1* genes. **a**, **b** Forward and reverse strand sequence of novel homozygous missense mutation c.863 G > Cin *DHH* gene (Patient-1). **c**, **d** Forward and reverse strand sequence of homozygous missense mutation c.1339 G > Ain *BBS1* gene (Patient-3). **e**, **f** Forward and reverse strand sequence of homozygous missense mutation c.1216 C > Tin *AP4B1* gene (Patient-5).

After genomic analysis of the WES data, we have identified five (5) unrelated patients with extremely rare pediatric genetic diseases carrying autosomal recessive pathogenic variants in different genes. Specifically, WES identified three homozygous variants: c.863 G > C (p.Pro288Arg), c.1339 G > A (p.Ala447Thr), and c.1216 C > T (p.Arg406Ter) in *DHH*, *BBS1*, and *AP4B1* genes, respectively (Table 1: Patient 1, Patient 3 and Patient 5). Our analysis also identified two compound heterozygous variants c.3503_3504delTC (p.Leu1168Glnfs*) and c.2972dupT (p.Met991Ilefs*) in *GNPTAB* (Table 1: Patient 2) as well as c.229 G > C(p.Gly77Arg) and c.792_793delAG(p.Arg264Serfs*) in *SURF1* (Table 1: Patient 4). All these mutations were further verified by Sanger sequencing (Figs. 1 and 2). Below are the results from clinical observation and the genetic mutations from WES.

**Table 1:** Summary of patients clinical information and list of clinically relevant mutations in a range of rare genetic diseases in Bangladesh.

Patient Id, Age, Consanguinity Status (Yes/No)	Clinical information	Supported disease on the basis of genotype and phenotype information	Gene name	RefSeq Id	Variant type	Variant	Zygosity	Clinical significance
1 (22 Y, No)	• primary amenorrhea • under development of breast • tanner staging revealed breast stage B2(Rt) &B3(Lt)• pubic hair stage 4 with female pattern• normal female external genitalia with no palpable gonads• absence of mularian derivatives,• testis in left inguinal region• karyotyping showed 46XY	46,XY gonadal dysgenesis (GD) or 46,XY sex reversal 7	*DHH*	NM_021044 **Exon 3**	Missense	c.863 G > C; p.Pro288Arg	Homozygous **(Novel)**	**Likely Pathogenic**
2 (6 M, No)	• respiratory distress• transient tachypnea of newborn• congenital heart disease (PDA 3 mm, small PFO)• prominent philtrum• coarse facis• widely spaced nipple• suspicion of joint contracture both knee and elbows	Mucolipidosis II alpha/beta (ML II)	*GNPTAB*	NM_024312 **Exon 19**	Frameshift Deletion	c.3503_3504delTC; p.Leu1168 Glnfs*	Heterozygous	**Pathogenic**
			*GNPTAB*	NM_024312 **Exon 15**	Frameshift Insertion	c.2972dupT; p.Met991Ilefs*	Heterozygous **(Novel)**	**Likely Pathogenic**
3 (8 Y 4 M, Yes)	• 8 episodes of focal seizure with impaired awareness• impaired intelligence• fatigue• language delay• constipation• hypothyroidism• polydactyly• obesity• strabismus	Bardet–Biedl Syndrome (BBS)	*BBS1*	NM_024649 **Exon 13**	Missense	c.1339 G > A; p.Ala 447Thr	Homozygous	**Likely Pathogenic**
4 (4 Y, No)	• cannot sit and walk with or without support• slurred speech• bilateral convergent squint• low muscle tone• lactic acidosis (8.9 mmol/L)• bilateral cerebral and cerebellum leukodystrophy	Leukodystrophy along Leigh syndrome	*SURF1*	NM_003172 **Exon 3**	Missense	c. 229 G > C; p.Gly77Arg	Heterozygous **(Novel)**	**Likely Pathogenic**
			*SURF1*	NM_003172 **Exon 8**	Frameshift deletion	c.792_793delAG; p.Arg264 Serfs*	Heterozygous	**Pathogenic**
5 (15 M, Yes)	• fever for 2 days with convulsion in the form of lip-smacking, rolling of eye ball lasted for more than 8 min• walk few steps with support• speech delay	Spastic paraplegia-47 (SPG-47)	*AP4B1*	NM_006594 **Exon 8**	Stopgain	c. 1216 C > T p.Arg406Ter	Homozygous	**Likely pathogenic**

Note: *Y* = Year; *M* = Month

### Patient 1

A 22-year-old girl, presenting with primary amenorrhea and underdevelopment of sexual characteristics, was referred for genetic evaluation. Her antenatal and peripartum history was uneventful. She was born by normal delivery and declared as a girl by a local physician, and raised as a female. Her cognitive, language, and motor developmental milestones were all normal, and her school performance was satisfactory in the mainstream setting. She was the third child of a non-consanguineous marriage, with no known family history of medical illness. At the age of 15 years, she failed to establish menses and consult with a local physician. Later, she was referred to NeuroGen Children’s Healthcare for clinical exome tests by the clinician at the department of Endocrinology & Metabolism, Bangabandhu Sheikh Mujib Medical University (BSMMU), Dhaka, Bangladesh at the age of 22 years. At that examination, her height was 164 cm, BMI 17.1 kg/m2, and arm span 186 cm. Tanner staging revealed breast stage B2(Rt) & B3(Lt), pubic hair stage 4 with female pattern, normal female external genitalia with no palpable gonads. Hormonal evaluation showed FSH 175.12 IU/L (normal range for<33 years menstruating women: <7.0 lU/L), LH 51.13 IU/L (Women, follicular phase of menstrual cycle: 1.68 to 15 IU/L; Women, midcycle peak: 21.9–56.6 IU/L and Women, luteal phase: 0.61 to 16.3 IU/L), serum testosterone 92.8 ng/dl (Adult women normal range: 8–60 ng/dl and adult men normal range: 240–950 ng/dl), testosterone after HCG stimulation test 45 ng/dl, TSH 2.31 uIU/ml (Female at age 18–29: 0.4–2.34 mIU/L and Male at age 18–30: 0.5–4.15 mIU/L, FT4 1.58 ng/dl (Normal range: 0.7–1.9 ng/dl), estrogen 77.5 pg/ml (In premenopausal females, normal range: 30 to 400 pg/ml; In postmenopausal females, normal range: 0–30 pg/ml and In males, normal range: 10–50 pg/ml), ACTH 22.6 pg/ml (Normal range for a blood sample taken early in the morning are 9–52 pg/ml), cortisol 637 nmol/L. USG of the abdomen showed absence of Mullerian derivatives. MRI of the abdomen revealed a testis in the left inguinal region, and karyotyping showed 46XY.

After performing WES, a homozygous variant c.863 C > G (Table 1: Patient-1) in exon 3 of *DHH* gene was found that causes Proline to Arginine change at position 288 which belongs to *DHh*-C functional domain (amino acids 199–396) of Desert Hedgehog protein. This variation was not observed in the gnomAD database (http://gnomad.broadinstitute.org) or the Human Gene Mutations Database (HGMD) (http://www.hgmd.org) or the 1000 Genomes Project and most likely reflects a novel mutation (see Fig. 2). Multiple in silico analysis tools PolyPhen-2^11^ and CADD_phred^12^ predict that the novel variant c.863 C > G is damaging with high probability. This mutation led to a nonconservative amino acid substitution, changing a highly conserved Proline residue to Arginine. This gene is functionally shown to be relevant to the phenotype and homozygous mutations (missense/nonsense/frameshift)within this domain were classified as pathogenic for similar phenotypic patients in ClinVar database. This reported homozygous mutation can be classified as likely pathogenic in accordance with ACMG criteria^13^. For the remainder of patients, we have used similar criteria for variant interpretations.

### Patient 2

Patient 2, a 6-month-old girl, was admitted to the hospital in respiratory distress, which had presented soon after birth. She was diagnosed with transient tachypnea of the newborn and was also noted to have congenital heart disease (PDA 3 mm, small PFO). She presented with craniofacial dysmorphology, including a prominent philtrum, coarse facial features, and widely spaced nipples. Her joint contractures of both knee and elbows were suspected to be the consequence of intrauterine growth restriction (IUGR). She was the firstborn of a non-consanguineous marriage and her paternal and maternal family members were healthy.

Following WES, a compound heterozygous mutation impacting *GNPTAB* was identified (c.3503_3504delTC [p.Leu1168Glnfs*], and c.2972dupT [p.Met991Ilefs*] (see Table 1: Patient 2). The maternally inherited frameshift deletion, c.3503_3504delTC (Fig. 1), causes a change Leucine to Glutamine at position 1168 of *GNPTAB* protein and leads to a premature termination codon 5 amino acids downstream. The variant is well described in the literature^14–17^ and is the most common pathogenic variant for *GNPTAB*-related disorders in several populations, usually associated with a severe phenotype. Due to the potential impact of frameshift variants and the reported evidence, this variant is classified as pathogenic for *GNPTAB*-related disorders. The paternally inherited heterozygous frameshift insertion variant, c.2972dupT (Fig. 1), was identified in the exon 15 of *GNPTAB* that causes Methionine to Isoleucine change at position 991 of the protein and leads to a premature termination codon 25 amino acids downstream. This variation is not observed in gnomAD browser (http://gnomad.broadinstitute.org), the Human Gene Mutations Database (HGMD) (http://www.hgmd.org) or the 1000 Genomes Project, and most likely reflects a novel variant. These novel compound (frameshifts) variants can be classified as ‘likely pathogenic’ as they meet the likely pathogenic ACMG criteria^13^.

### Patient 3

Patient 3, an 8 year-4-month-old boy, was referred to the pediatric neurology clinic due to focal seizures with loss of consciousness occurring 8 times within a 12 months period. His cognitive and language milestones were all delayed, and he also presented with constipation, and obesity. On examination, polydactyly and strabismus were noted, and initial workup revealed hypothyroidism. He was the second born of a consanguineous marriage and his older sister was healthy.

In this patient, a rare homozygous mutation impacting *BBS1*gene was identified using WES (c.1339 G > A[p.Ala447Thr])that causes Alanine to Threonine change at position 447 of *BBS1* protein. This gene is a known cause of Bardet–Biedl Syndrome (BBS), an autosomal recessive and genetically heterogeneous ciliopathy characterized by retinitis pigmentosa, obesity, kidney dysfunction, polydactyly, behavioral dysfunction, and hypogonadism^18^. In very rare cases, seizure has also been found in patients of BBS^19,20^. The variant c.1339 G > A falls at the last nucleotide of exon 13 of the *BBS1* coding sequence, which is part of the consensus splice site for this exon. Nucleotide substitutions within the consensus splice site are relatively common causes of aberrant splicing^21^. This variant was not found in the Genome Aggregation Database (gnomAD) as homozygous state. The heterozygous state of the variant is present in population databases at extremely rare frequencies (rs200116631, gnomAD frequency = 0.00003). Patient 3 meets the major clinical features of BBS with regard to hypothyroidism, polydactyly, obesity, strabismus, impaired intelligence in comparison to peers, and language delay. Therefore, this variant can be classified as ‘likely pathogenic’ in accordance with ACMG guideline. This variant was not screened in the parents’ DNA (Fig. 2).

### Patient 4

A 4-year old boy presented to the hospital with parental concerns about loss of motor skills. Specifically, they described a one-month history of difficulty sitting, standing, and walking. According to his mother, he was able to stand, and then walk, albeit unsteadily, by 29 months. His language development followed a similar regressive pattern: he was using some simple one and two syllable words used in the appropriate context by 19 months but subsequently his speech became slurred and dysarthric at 29 months. On examination, when he first presented at 48 months, he was alert and responsive, with intact vision and hearing and a bilateral convergent squint. He had normal reflexes but low muscle tone and bilateral weakness in all muscle groups. No abnormal or involuntary movements were noted. His OFC was 46 cm. Biochemical findings were random blood sugar of 4.7 mmol/L, serum ammonia and lactic acid level was 45 µmol/L and 8.9 mmol/L (Lactic acidosis) respectively. CT Scan of his brain identified bilateral cerebral and cerebellum leukodystrophy/ischemic change. He was the second child of a nonconsanguineous marriage and his elder brother was healthy.

A compound heterozygous variant in *SURF1*gene was identified using WES (c.229 G > C[p.Gly77Arg] andc.792_793delAG[p.Arg264Serfs*27]). Mutations in this gene are a cause of Surf1 protein deficiency, a recessively inherited severe mitochondrial neurological disorder, and is the most frequent cause of Leigh syndrome (LS) associated with cytochrome c oxidase (COX, complex IV) deficiency^22,23^. The maternally inherited heterozygous missense variant c.229 G > C [p.Gly77Arg] (Fig. 1) causes a Glycine to Arginine change at codon 77 of the *Surf1* protein. The heterozygous missense variant is located in the N-terminal domain (61-79 amino acid) of the Surf1 protein that might disrupt the assembly of the *surf1* protein in the mitochondrial inner membrane. This variation was not observed in gnomAD database (http://gnomad.broadinstitute.org) or the Human Gene Mutations Database (HGMD) (http://www.hgmd.org) or the 1000 Genomes Project and likely reflects a novel mutation. In silico analysis tools SIFT_score^24^, PolyPhen-2^11^, and CADD_phred^12^ predict that the variant is likely to be pathogenic (Table 1). This variant results in a nonconservative amino acid substitution, changing a highly conserved residue Glycine to Arginine. This novel variant is defined as likely pathogenic as it meets the criteria of ACMG likely pathogenic variant^13^. The p.Gly77Arg missense variant was in trans with another confirmed deleterious mutation, p.Arg264Serfs*. This paternally inherited frameshift deletion, c.792_793delAG (Fig. 1), causes a change Arginine to Serine at position 264 of the Surf1 protein that leads to a premature termination codon 27 amino acids downstream (p.Lys291Ter) in the new reading frame. This variant has been reported previously in a patient with Leigh syndrome who also harbored a second *SURF1* variant^25^ and has also been reported in a homozygous state in multiple individuals with Leigh syndrome^22,26^. This gene functionally relevant to the phenotype and ClinVar^27^ contains an entry for this variant as ‘pathogenic’ (Variation ID: 215238).

### Patient 5

Patient 5, a 15-month old boy born to consanguineous healthy parents, was referred for genetic testing after being admitted to the hospital with an 8-min tonic-clonic seizure in the context of a 48-h fever. Prior to this, there had been no major concerns. He was delivered by an uneventful LSCS following a normal pregnancy and without any perinatal complications. His birth weight was 2.5 kg. He was able to hold up his head unsupported at 3 months, sit upright independently at 10 months, and by 15 months was able to walk a few steps with support. His speech was delayed, however, and he was only using one and two-syllable words such as “baba”, “mama”, and “dada”. On examination, at the time of first admission, he was conscious, yet irritable, and his vital signs were within the reference range. His muscle bulk was normal, as were muscle tone and reflexes. He had a plantar-extensor response. His EEG showed some non-specific background polymorphic activity. Brain MRI examination showed minimal atrophy in both fronto-parietal and temporal lobes. Serum ammonia and lactic acid level were 60.0 µmol/L and 1.3 mmol/L respectively. He was the second-born, with his older female sibling, now age 6 years, previously diagnosed with psychomotor and language delay.

Using WES, a*AP4B1*-impacting homozygous variant c.1216 C > T (p.Arg406Ter) was identified that alters an amino acid from Arginine to stop codon, p.Arg406Ter.The *AP4B1* gene encodes a 739-amino acids beta 1 subunit of the heterotetrameric adaptor protein (AP) complex^28^. Abnormality of this gene leads to spastic paraplegia-47 (SPG47), intellectual disability, or an AP-4 deficiency syndrome^29–31^. The variantc.1216 C > T is predicted to cause loss of normal protein function either through protein truncation or nonsense-mediated mRNA decay. It is a very rare allele; the allele frequency of this variant in gnomAD (http://gnomad.broadinstitute.org) is 0.00005567 (14/251462). These 14 alleles are present as heterozygotes and no homozygotes were observed in gnomAD. There is also no entry of this variant in 1000 g database, indicating it is not a common variant. ClinVar^27^ has two submissions for this variant (Variation ID: 422147), which were listed as likely pathogenic. This variant was not screened in the parents’ DNA (Fig. 2).

## Discussion

In this study, we performed WESin a cohort of five (5) unrelated patients with rare genetic diseases together with analysis of biological processes to explore the implementation of whole-exome sequencing for diagnosis of rare genetic diseases in Bangladeshi children. Bangladesh is a small and densely populated developing country with limited healthcare resources. As a result, there is limited availability of targeted genetic tests for diagnosing a broad range of genetic disorders. WES provides a comprehensive front-line genomic test to efficiently diagnosis a broad range of rare genetic disorders. The five patients described here had complex phenotypic manifestations. Therefore, it was difficult to do targeted genetic testing with diagnosis-specific customized panels. In all five (5)patients, the mutations described are very likely to be etiologically related to the clinical pictures described.

Patient 1 presented with primary amenorrhea and underdevelopment of secondary sexual characteristics with a 46XY karyotype (Table 1). From our genetic analysis, we identified a homozygous variant c.863 C > G (Table 1: Patient-1) in exon 3 of *DHH* that causes Proline to Arginine change at position 288 of Desert Hedgehog protein. *DHH* gene consists of 3 exons and is located on chromosome band 12q13.12^32^. It encodes a Desert Hedgehog signaling molecule that consists of 2 domains: a soluble amino-terminal “Hedge” domain (*DHh*-N: 23-198 amino acid) and a carboxyl-terminal “Hog” domain (*DHh*-C: 199 to 396 amino acid)^33^. The Hedgehog pathway plays a critical role in developmental processes, embryogenesis, cell proliferation and tissue regeneration^33–36^. *DHH* is important in gonadal differentiation^37–39^ as it is produced by Sertoli cells and is important for fetal Leydig cell differentiation^40^. Once cleaved, *DHh*-N domain functions as a signaling molecule while the *DHh*-C participates in autoprocessing regulation^34^. Disruption of the *DHh* auto-processing due to clinically relevant variants is associated with 46,XY gonadal dysgenesis (GD) or 46,XY sex reversal 7^38,39,41^. We speculate that the p.Pro288Arg variant diminishes autoproteolysis activity of the *DHh*-C domain of *DHH* protein that lead to the clinical condition 46,XY gonadal dysgenesis (GD) or 46,XY sex reversal 7 in our patient (Table 1). Other clinical descriptions of mutations in this gene are also reported like polyneuropathy along with GD that is mostly associated with the complete disruption of*DHH* another domain *DHh*-N^42^. Patient1 did not exhibit this feature as the variant is situated in the *DHh*-C domain.

Patient 2 presented with respiratory distress, congenital heart disease, craniofacial dysmorphology, and joint contractures (Table 1). A compound heterozygous mutation was identified (one novel heterozygous allele of frameshift insertion and another heterozygous allele of frameshift deletion) in exons 15 and 19 of *GNPTAB*, respectively(Table 1: Patient-2). The frameshift deletion, c.3503_3504delTCis well described in the literature^14–17^ and is the most common pathogenic variant for *GNPTAB*-related disorders in several populations, and is usually associated with a severe phenotype. According to the Kudo et al. and Plante et al. this variant can cause a deleterious effect on *GNPTAB*gene resulting in Mucolipidosis II (MLII) phenotype in the probands. They concluded that the presence of a homozygous 3503_3504delTC variant is sufficient to produce MLII^43,44^. Tappino et al. reported that its allele frequency is 51.1% (47/92) in their MLI and MLIII alpha/beta patients from diverse ethnic backgrounds (Italy, Argentina, Bangladesh, Bulgaria, and Hungary)^16^. In addition, this variant was found worldwide and resulted most probably from a unique founder molecular lesion^14,44^. Functional studies demonstrate that the variant produces a truncated protein that is retained in the endoplasmic reticulum and not transported to the Golgi, and therefore does not form a mature subunit^17^. This variant has also been shown to result in absent or significantly decreased enzyme activity in patient fibroblasts^43,45,46^.

In this patient, another heterozygous frameshift insertion variant, c.2972dupT, was identified in exon 15 of *GNPTAB* that causes Methionine to Isoleucine change at position 991 of protein and leads to a premature termination codon 25 amino acids downstream. We hypothesize that this novel truncating mutation, p.Met991Ilefs*, along with another truncating frameshift mutation, p.Leu1168Glnfs*, is sufficient to result in MLII. A single cytosine insertion at codon 1,049 has been identified in a homozygous state in a male ML II patient resulting in a frameshift and a premature termination (p.Gly1049Argfs*16)^47^, and the insertion of the homologous mutation in the murine *Gnptab* results in mice displaying the clinical and biochemical features of ML II disease including the complete loss of GlcNAc-1-phosphotransferase activity^48^. Mucolipidosis II-related pathogenic variants, p.Gly1049Argfs* and p.Leu1168Glnfs*, inhibit the exit from the endoplasmic reticulum and proteolytic cleavage of GlcNAc-1-phosphotransferase precursor protein (*GNPTAB*)^17^. Almost all patients with classical severe MLII have nonsense or frameshift mutations in *GNPTAB*^43,44,47,49–51^. From the patient’s genetic data, clinical conditions, and the above-mentioned review of literature, we confirm that patient 2 is likely to be affected by Mucolipidosis II alpha/beta (MLII) (Table 1).

Patient 3 was presented with focal seizures, delayed cognitive and language milestones, hypothyroidism, polydactyly, obesity, and strabismus (Table 1). On WES, a rare homozygous mutation (c.1339 G > A[p.Ala447Thr]) impacting *BBS1* gene was identified. At least 19 genes are associated with BBS^52^, with *BBS1* gene being the most frequently affected in BBS patients^53^. *BBS1* maps to chromosome 11q13.1 and is composed of 17 exons^53^, which encode a protein of 593 amino acids. The N-terminal part shares homology with other BBS-associated gene products (*BBS2* and *BBS7*). Mutations in *BBS1* gene are the most common cause of BBS, accounting for about one-quarter of all cases. The most common pathogenic sequence alterations in *BBS1* is the missense mutation p.M390R^53^ and is found in about 20–30% of BBS cases^54,55^. Schmid et al. found a homozygous variant c.479 G > A in the last nucleotide (splice site mutation) of exon 5 of *BBS1* that causes aberrant splicing of *BBS1* transcripts and co-segregated with the phenotype of retinitis pigmentosa^56^. Although, the clinical presentation of this patient is consistent with Bardet–Biedl Syndrome (BBS), given the vast number of loci associated with BBS (*BBS1-BBS21*) combined with the fact that no customized BBS targeted gene panel exists in Bangladesh, a decision was therefore taken to undertake WES. From the WES data, we analyzed all causal genes for BBS, but did not find any clinically relevant variants in those genes except for the c.1339 G > A variant in *BBS1*. Patient 3 meets the major clinical features of BBS with regard to hypothyroidism, polydactyly, obesity, strabismus, impaired intelligence in comparison to peers, and language delay. Therefore, we define the variant as likely pathogenic (criteria described in patient 1) and assume that it is associated with BBS (Table 1). Our patient also has a history of seizures which is rare among BBS patients but has been previously reported in some BBS patients^19,20^.

Patient 4 was presented with loss of cognitive and motor skills, lactic acidosis, and bilateral cerebral and cerebellum leukodystrophy (Table 1). On WES, a compound heterozygous variantin *SURF1* gene was identified (c.229 G > C[p.Gly77Arg] andc.792_793delAG[p.Arg264Serfs*]) (Table 1: Patient-4).The *SURF1* gene encodes a 300 amino acid long protein that contains two predicted transmembrane domains and acts as an important factor in cytochrome c oxidase (COX) assembly and maintenance. COX is the terminal component of the mitochondrial respiratory chain that mediates the transfer of electrons to oxygen^57,58^. In multiple literatures, it was established that missense pathogenic variants in *SURF1*is associated with deficient cytochromecoxidase assembly in Leigh syndrome patients^59,60^. *SURF1*-Associated Leigh Syndrome occurs due to null mutations accounts for 86% and missense mutations accounted for 14% of the disease alleles^61^. We hypothesize that this missense variant will produce an unstable *surf1* protein that will be readily degraded. In addition, p.Gly77Arg missense variant is in trans configuration with another definitive deleterious variant, p.Arg264Serfs*. Teraoka et al. identified a missense variant c.820 T > G along with the deleterious variant p.Arg264Serfs* in a Japanese patient with Leigh syndrome (256000). Most of the clinical conditions of this patient are consistent with the clinical symptoms present in our patient (patient-4)^25^. CT Scan of the patient’s brain found bilateral cerebral and cerebellum leukodystrophy or ischemic change. A similar pattern of leukodystrophy was also found in a child carrying a homozygous variant, p.Arg264Serfs*, in *SURF1*. Most of the clinical conditions of this patient are also consistent with the clinical symptoms present in our patient (Patient-4)^62^. From the patient’s genotype (Patient-4), phenotype, and the above-mentioned review of literature, we hypothesize that the compound heterozygous variant p.Gly77Argand p.Arg264Serfs* are associated with this patient’s phenotype (Table 1).

Patient 5 presented with tonic-clonic seizures in the context of a 48 h fever. On WES, a homozygous nonsense variant c.1216 C > Tin the exon 8 of *AP4B1* gene was identified that alters an amino acid from Arginine to the stop codon, p.Arg406Ter.*AP4B1* encodes a 739-amino acids beta 1 subunit of the heterotetrameric adaptor protein (AP) complex^28^. AP-complex is ubiquitously expressed in neurons throughout the embryologic and postnatal developmental stages and interacts with delta2 and α-amino-3-hydroxy-5-methyl4-isoxazolepropionic acid (AMPA) glutamate receptors to selectively transport them from the trans-Golgi network to the postsynaptic somatodendritic domain^63^. The vast majority of pathogenic variants identified in this gene are truncating variants, such as nonsense variants, frameshifts, and canonical splice sites which often disrupt gene function by leading to a complete absence of the transcript through nonsense-mediated decay^64^. These mutations result in a non-functional AP-4 complex that leads to abnormal brain growth and development, which are associated with spastic paraplegia-47 (SPG47), intellectual disability, or AP-4 deficiency syndrome^29–31^.

To date, twenty-two (22) pathogenic variants in *AP4B1* have been reported^64^ which includes the c.1216 C > T variant found in our patient. This variant was reported in two unrelated *AP4B1*-associated SPG47 patients as compound heterozygotes^31^. Both were female and the disease was diagnosed at the age of 29 months and 36 months, respectively. The two patients had speech delay, infantile hypotonia, delayed motor development, developmental delay/intellectual disability, seizures, and lower extremity spasticity, but did not present with microcephaly. Our patient is clinically similar with regard to the seizures, absence of microcephaly, delayed speech and motor development. Spastic paraplegia was not seen in this patient, as this typically develops later in the course of the disease^31,64^. As the patient’s phenotype matches with other patients described in the literature, we hypothesize that the homozygous mutation, c.1216 C > T, is associated with Spastic Paraplegia-47 (Table 1).

Our findings demonstrate the potential utility of WES as a first-line diagnostic approach in patients lacking a clear differential diagnosis in a developing country with limited healthcare resources. This approach will facilitate the identification of rare genetic diseases, accelerate rare disease research and ultimately help implement precision medical care^65^. We appreciate that genetic testing may not directly impact the course of the disorder, which, for neurodevelopmental disorders at least, is often chronic and without disorder-modifying treatments. However, there are a number of ways in which genetic testing can impact care pathways, which is true in Bangladesh to the same extent as other countries. Specifically, families generally seek genetic testing as it provides them with a sense of understanding regarding the origin of their child’s difficulties. It also avoids the child going though unnecessary diagnostic investigations (diagnostic odyssey). It also opens up the opportunity of expert counseling for the family regarding prognosis and other possible medical complications, which also offers to possibility of screening and early intervention. We, therefore, believe that even in a country with limited health resources there are many benefits to diagnostic testing.

## Methods

### Study cohort

From March 2019 to February 2020, 280 unrelated patients with neurodevelopmental disorders (NDDs), cancer, and rare genomic diseases were referred to NeuroGen Children’s Healthcare from multiple tertiary hospitals within the country. Of the 280 cases, 52 patients were referred for targeted cancer panel sequencing by their physician^4^. The remaining patients were suspected of rare developmental disorders and were referred for WES. In each patient, the treating medical team noted a range of symptomatic manifestations, across different systems. The physicians collected phenotypic details, as summarized in Table 1. The children were not amenable to further formal clinical evaluation of development and cognition, and their families did not consent to further investigations due to the recent pandemic (COVID19).

### Ethics statement

The study protocol was reviewed and approved by the Ethical Review Committee of the Faculty of Biological Sciences, University of Dhaka, and Institutional Review Board of Holy Family Red Crescent Medical College. Before participant enrollment, written informed consent including the use of peripheral blood and clinical data for research use and publication were obtained from the parents.

### DNA extraction

Peripheral blood samples were collected from participants in an EDTA vacutainer tube. Genomic DNA was isolated using ReliaPrep™ Blood gDNA isolation kit (Promega, USA) according to manufacturer instructions. The concentration and quality of DNA was determined using NanoPhotometer C40 (Implan, Germany) (Supplementary Table 1). The quality of DNA was also assessed using 0.8% agarose gel electrophoresis.

### Whole exome sequencing

Whole exome sequencing (WES) was conducted using the HiSeq sequencing platform. DNA libraries were prepared using the SureSelect XT library preparation kit and the SureSelect V7-Post Target Enrichment Kit. HiSeq uses a Burrows–Wheeler Aligner (BWA)^66^ and the Genome Analysis ToolKit (GATK 4.0.11.0)^67^ for converting raw sequence reads to Binary Alignment Map (BAM) and Variant Call Format (VCF) v4.1 files. The DNA sequence was assembled and aligned to reference gene sequences based on the human genome build GRCH38/UCSC hg38. ANNOVAR (2018Apr16 version) was used for functional annotation of the variants. For genomic annotations, GenomeArc (http://genomearc.com/), a custom clinical genetic annotation tool, was used to annotate gnomAD variant frequencies, clinvar pathogenic mutations, and pLI. Variant classification analysis was conducted based on the American College of Medical Genetics (ACMG) guidelines^13^ and Sanger sequencing was used to confirm the variants^68^.

### Variant validation

The recessive variants identified from WES were validated by Sanger sequencing using standard protocols. Validation primers (Supplementary Table 2) were designed using Primer 3 plus software, IDT, and UCSC Genome Browser. Primer and PCR conditions are included in the supplementary file (Supplementary Tables 2 and 3, respectively). The PCR products were run on 2.0% agarose gel and the products then purified using the Wizard^®^ SV Gel and PCR Clean-Up System (Promega, USA) according to the manufacturer’s instructions. Cycle sequencing was performed using purified PCR products as templates and BigDye^®^ Terminator v3.1 (Applied Biosystem, USA). Bidirectional Sanger sequencing was performed using 3500 DNA Analyzer (Applied Biosystem, USA) to determine the sequence. Sanger sequencing data were analyzed using Sequence Scanner v2.0 (Applied Biosystem, USA).

### Reporting summary

Further information on research design is available in the Nature Research Reporting Summary linked to this article.

## Supplementary information

Supplementary Information

Reporting Summary

## Data Availability

Due to Bangladesh data privacy laws regarding patient data, both this and our patient consent does not permit the genomic or phenotypic data to be made publicly available. Genomic and phenotypic data used and/or analyzed during the current study can be shared for any collaborative research that involves NeuroGen Children’s Healthcare. Please request this via the corresponding author
