# Supplementary material for: Whole exome sequencing uncovered highly penetrant recessive mutations for a spectrum of rare genetic pediatric diseases in Bangladesh
Source: NPJ Genom Med. 2021 Feb 16;6:14. doi: 10.1038/s41525-021-00173-0 (PMC7887195; doi:10.1038/s41525-021-00173-0)
Supplement: Supplementary file 1 — Supplementary Information [file 41525_2021_173_MOESM1_ESM.pdf]

Supplementary Information: Whole exome sequencing uncovered highly penetrant recessive mutations for a spectrum of rare genetic pediatric diseases in Bangladesh

Supplementary Table 1: Biospecimen Description (DNA)

Case ID	Gender	Conc in ng/µl	260/280 ratio	260/230 ratio
1	Female	49.75	1.83	2.09
2	Female	209.8	1.90	2.28
3	Male	117.55	1.91	2.3
4	Male	104.4	1.89	2.11
5	Male	142.2	1.92	2.4

Supplementary Table 2: Details of Sanger sequencing validation primers

Case ID	FP Id.	FP Sequence	RP Id.	RP Sequence	Product Size
1.	DHH_pP288R_F	CGAGAGTACCAA TGCATGCC	DHH_pP288R_R	GCTTCATTTGTG GCTGTGGA	700
2.	GNPTAB_pK 1168Qfs*5_F	AAGAATCATTGT ACCCAGGAG	GNPTAB_pK 1168Qfs*5_R	ATAGAATATCA TTCCCCCAGAG	529
	GNPTAB_pM 1168Ifs*25_F	AGACTAGCTATG AATACCCACAG	GNPTAB_pM 1168Ifs*25_R	ACTAAGGCTAC TTGAAATGTGC	849
3.	BBS1_pA447T_F	GCAGATTGTTTG GGGAAGAA	BBS1_pA447T_R	TGGATTTGCAG AGGTGAGTG	381
4.	SURF1_ pG77R_F	GTTGAACTCAAG TAAAACAGGC	SURF1_ pG77R_R	GAGCCCTAGAT CCCACTTAC	557
	SURF1_ pR264 Sfs*27_F	CCAGGATTTTAT GATGAACCAG	SURF1_ pR264 Sfs*27_R	GAATACTGTCCT TCCTCCTAAC	629
5.	AP4B1_pR238X_F	GGTGGCAATCAA TTCGAAGA	AP4B1_pR238X_R	CACACCTCCAA AACCCATTT	359

Supplementary Table 3: PCR Condition

Temperature	Time	Cycles
95 ℃	5 min	
95 ℃	30 sec	35
60 ℃	30 sec	
72 ℃	1 min	
72 ℃	7 min	
4 ℃	storage	
